# Supplementary material for: Lactic Starter Dose Shapes S. aureus and STEC O26:H11 Growth, and Bacterial Community Patterns in Raw Milk Uncooked Pressed Cheeses
Source: Microorganisms. 2021 May 18;9(5):1081. doi: 10.3390/microorganisms9051081 (PMC8157849; doi:10.3390/microorganisms9051081)
Supplement: Supplementary file 1 [file microorganisms-09-01081-s001.zip › microorganisms-1202065-supplementary.pdf]

Table S1. Biochemical and microbial characteristics in the raw milk used (*n* = 3). The differences according to the farm were estimated by a Wilcoxon's test (*n* = 6). **p* < 0.05; ***p* < 0.01; ****p* < 0.001; tendency; ns non-significant. NC Non calculable because under the detection threshold by plate counting [a] < 10 UFC/mL; [b] < 5 UFC/mL.

		Mean F_15	SD F_15	Mean F_38	SD F_38	Mean	SD	Farm effect
Biochemical characteristics of raw milks	pH	6.68	0.02	6.65	0.02	6.66	0.02	0.268 (ns)
	Dry matter (%)	12.33	0.31	12.77	0.35	12.55	0.38	0.268 (ns)
	Lactose (g/L)	50.70	0.66	50.57	0.81	50.63	0.66	0.825 (ns)
	Fat (g/L)	37.5	2.3	39.0	3.8	38.2	2.9	0.507 (ns)
	Protein (g/L)	34.2	1.0	34.9	2.5	34.6	1.7	1.000 (ns)
	Fat/Protein ratio	1.09	0.07	1.12	0.12	1.11	0.09	1.000 (ns)
	Urea (mg/L)	208	13	197	15	203	14	0.506 (ns)
	Somatic cells (/mL)	233667	167506	227333	338400	230500	238833	0.700 (ns)
	casein (g/L)	31.19	0.94	31.78	2.25	31.49	1.58	0.825 (ns)
	Lipolysis (mEq/100mg of fat)	0.18	0.09	0.28	0.13	0.23	0.11	0.700 (ns)
Fatty acids profiles in raw milks	total unsaturated (g/L)	10.11	0.52	10.29	0.41	10.20	0.43	0.400 (ns)
	mono-unsaturated (g/L)	8.02	0.78	8.02	0.49	8.02	0.58	1.000 (ns)
	poly-unsaturated (g/L)	1.18	0.10	1.19	0.10	1.18	0.09	1.000 (ns)
	total saturated (g/L)	25.49	1.92	26.71	3.34	26.10	2.52	0.700 (ns)
Microbial characteristics of raw milks	Total germs (CFU/mL)	$1.30x10^4$	$1.73x10^3$	$6.33x10^3$	$1.53x10^3$	$9.67x10^3$	$3.93x10^3$	0.077 (.)
	coagulase + *Staphylococcus* (CFU/mL)	$1.04x10^2$	$1.52x10^2$	$6.16x10^2$	$1.03x10^3$	$3.60x10^2$	$7.18x10^2$	0.700 (ns)
	E. coli (CFU/mL)	NC[a]	NC[a]	NC[a]	NC[a]	NC[a]	NC[a]	NC[a]
	STEC (CFU/mL)	NC[b]	NC[b]	NC[b]	NC[b]	NC[b]	NC[b]	NC[b]
Pathogens levels in inoculated milks	*S.aureus*	$9.30x10^2$	$5.47x10^2$	$6.37x10^2$	$5.11x10^1$	$7.84x10^2$	$3.82x10^2$	0.886 (ns)
	STEC O26:H11	$1.04x10^2$	$2.13x10^1$	$1.13x10^2$	$2.64x10^1$	$1.08x10^2$	$2.21x10^1$	1.000 (ns)

Table S2. pH values, dry matter content (%), coagulase + S. aureus and STEC O26:H11 levels (CFU/g) and Young's modulus values (MPa) in the inoculated and control cheeses at D0.25 (pH only) D1 and D28. The differences according to the lactic starter dose were estimated by Kruskal-Wallis' tests (n=6), and according to the farm by Wilcoxon's tests (n=6). **p* < 0.05; ***p* < 0.01; ****p* < 0.001; . tendency; ns non-significant. NC[a] Non calculable because under the detection threshold by plate counting; NC[b] Non calculable because of only one available value; NC[c] Non calculable because of too small number of available values; [d] only tested with data from the F_38.

			x0,1-Dose				x0,1-Dose		x1-Dose				x1-Dose		x2-Dose				x2-Dose		Dose Effect	Farm Effect	Dose x Farm	Time Effect	Time x Dose	Time x Farm	Time x Dose x Farm	Inoculation Effect
			Mean F_15	SD F_15	Mean F_38	SD F_38	Mean	SD	Mean F_15	SD F_15	Mean F_38	SD F_38	Mean	SD	Mean F_15	SD F_15	Mean F_38	SD F_38	Mean	SD								
pH values	Inoculated cheeses	D0,25	6.54	0.06	6.51	0.02	6.53	0.04	5.93	0.17	5.93	0.14	5.93	0.14	5.74	0.17	5.74	0.05	5.74	0.11	0,002 (**)	0,859 (ns)	0,961 (ns)	3,17x10^{-8} (***)	5,10x10^{-13} (***)	0,021 (*)	0,351 (ns)	0,722 (ns)
		D1	5.36	0.12	5.58	0.11	5.47	0.16	5.24	0.08	5.23	0.04	5.23	0.05	5.17	0.05	5.22	0.04	5.19	0.04	0,006 (**)	0,413 (ns)	0,077 (.)					
		D28	5.16	0.05	5.32	0.02	5.24	0.10	5.23	0.03	5.38	0.04	5.30	0.09	5.24	0.04	5.40	0.04	5.32	0.10	0,335 (ns)	3,82x10^{-4} (***)	0,912(ns)					
	Control cheeses	D0,25	6.51	0.03	6.49	0.01	6.50	0.02	5.79	0.18	5.94	0.21	5.86	0.19	5.68	0.09	5.82	0.12	5.75	0.13	0,003 (**)	0,566 (ns)	0,469 (ns)	6,49x10^{-8} (***)	3,27x10^{-10} (***)	0,708 (ns)	0,119 (ns)	
		D1	5.38	0.21	5.64	0.16	5.51	0.22	5.20	0.08	5.26	0.03	5.23	0.06	5.17	0.04	5.20	0.04	5.19	0.04	0,009 (**)	0,184 (ns)	0,217 (ns)					
		D28	5.21	0.04	5.30	0.06	5.26	0.06	5.21	0.06	5.43	0.04	5.32	0.13	5.22	0.04	5.36	0.02	5.29	0.08	0,602 (ns)	0,001 (***)	0,048 (*)					
Dry matter content	Inoculated cheeses	D1	50.73	1.12	50.80	0.92	50.77	0.91	51.53	0.51	52.00	0.26	51.77	0.45	52.07	0.40	51.47	1.04	51.77	0.78	0,062 (.)	1,000 (ns)	0,510 (ns)	0,014 (*)	0,013 (*)	0,350 (ns)	0,873 (ns)	0,624 (ns)
		D28	50.20	0.36	51.57	0.40	50.88	0.82	53.50	0.85	54.37	1.16	53.93	1.03	54.90	1.66	54.80	2.69	54.85	2.00	0,004 (**)	0,508 (ns)	0,676 (ns)					
	Control cheeses	D1	51.07	0.45	50.10	0.92	50.58	0.84	52.43	0.67	51.73	1.04	52.08	0.87	52.10	0.87	51.67	1.29	51.88	1.01	0,040 (*)	0,121 (ns)	0,881 (ns)	9,79x10^{-5} (***)	0,729 (ns)	0,633 (ns)	0,735 (ns)	
		D28	51.27	0.38	51.07	1.02	51.17	0.70	53.07	1.01	53.13	2.25	53.10	1.56	53.63	1.46	52.73	0.45	53.18	1.09	0,015 (*)	0,691 (ns)	0,796 (ns)					
coagulase + S. aureus levels	Inoculated cheeses	D1	1.30x10^{6}	2.11x10^{6}	2.60x10^{6}	2.94x10^{6}	1.95x10^{6}	2.40x10^{6}	2.74x10^{5}	3.87x10^{5}	3.45x10^{5}	4.56x10^{5}	3.09x10^{5}	3.80x10^{5}	1.17x10^{5}	1.51x10^{5}	1.34x10^{5}	1.12x10^{5}	1.25x10^{5}	1.19x10^{5}	0,182 (ns)	0,258 (ns)	0,711 (ns)	0,003 (**)	0,122 (ns)	0,558 (ns)	0,746 (ns)	NC[c]
		D28	9.83x10^{4}	3.33x10^{4}	2.07x10^{5}	1.54x10^{5}	1.53x10^{5}	1.16x10^{5}	1.50x10^{4}	4.00x10^{3}	3.07x10^{4}	1.59x10^{4}	2.28x10^{4}	1.35x10^{4}	2.27x10^{4}	5.77x10^{3}	2.40x10^{4}	1.08x10^{4}	2.33x10^{4}	7.79x10^{3}	0,003 (**)	0,331 (ns)	0,334 (ns)					
	Control cheeses	D1	< 1000	NC[a]	2.27x10^{6}	3.71x10^{6}	NC[b]	NC[b]	3.16x10^{5}	4.44x10^{5}	1.88x10^{5}	2.87x10^{5}	2.39x10^{5}	3.09x10^{5}	< 100	NC[a]	7.42x10^{4}	1.00x10^{5}	NC[b]	NC[b]	0,404 (ns)[d]	NC[c]	NC[c]	NC[c]	NC[c]	NC[c]	NC[c]	
		D28	< 1000	NC[a]	3.70x10^{5}	NC[b]	NC[b]	NC[b]	< 100	NC[a]	9.00x10^{3}	NC[b]	NC[b]	NC[b]	< 100	NC[a]	3.95x10^{3}	NC[b]	NC[b]	NC[b]	NC[c]	NC[c]	NC[c]					
STEC O26:H11 levels	Inoculated cheeses	D1	1.39x10^{7}	7.77x10^{6}	4.11x10^{7}	6.40x10^{6}	2.75x10^{7}	1.62x10^{7}	3.35x10^{5}	1.44x10^{5}	1.22x10^{6}	1.76x10^{6}	7.75x10^{5}	1.21x10^{6}	1.10x10^{5}	4.94x10^{4}	2.82x10^{5}	3.36x10^{5}	1.96x10^{5}	2.35x10^{5}	0,002 (**)	0,605 (ns)	NC[c]	0,696 (ns)	0,529 (ns)	0,863 (ns)	0,952 (ns)	NC[c]
		D28	1.90x10^{7}	2.35x10^{7}	4.98x10^{7}	1.65x10^{7}	3.44x10^{7}	2.48x10^{7}	2.12x10^{5}	1.78x10^{5}	6.28x10^{5}	6.42x10^{5}	4.20x10^{5}	4.79x10^{5}	6.16x10^{4}	4.11x10^{4}	1.58x10^{5}	1.48x10^{5}	1.10x10^{5}	1.10x10^{5}	0,001 (**)	0,340 (ns)	NC[c]					
	Control cheeses	D1	<100	NC[a]	< 1000	NC[a]	NC[a]	NC[a]	< 100	NC[a]	<100	NC[a]	NC[a]	NC[a]	< 100	NC[a]	<100	NC[a]	NC[a]	NC[a]	NC[c]	NC[c]	NC[c]	NC[c]	NC[c]	NC[c]	NC[c]	
		D28	< 100	NC[a]	< 1000	NC[a]	NC[a]	NC[a]	< 100	NC[a]	< 100	NC[a]	NC[a]	NC[a]	< 100	NC[a]	< 100	NC[a]	NC[a]	NC[a]	NC[c]	NC[c]	NC[c]					
Young's modulus	Control cheeses	D28	0.313	0.401	0.285	0.132	0.299	0.298	0.312	0.316	0.445	0.249	0.377	0.290	0.358	0.388	0.470	0.223	0.414	0.318	0.006 (**)	3.31x10^{-6} (***)	0.420 (ns)					

Table S3. Microbial counts (CFU/g) at D28 in the control cheese cores and rinds. The differences according to the lactic starter dose were estimated by Kruskal-Wallis' tests (*n* = 6), and according to the farm by Wilcoxon's tests (*n* = 6). **p* < 0.05; ***p* < 0.01; ****p* < 0.001; . tendency; ns non-significant. NC[a] Non calculable because of only one available value.

		x0.1-Dose				x0.1-Dose		x1-Dose				x1-Dose		x2-Dose				x2-Dose		Dose Effect	Farm Effect	Dose x Farm
		Mean F_15	SD F_15	Mean F_38	SD F_38	Mean	SD	Mean F_15	SD F_15	Mean F_38	SD F_38	Mean	SD	Mean F_15	SD F_15	Mean F_38	SD F_38	Mean	SD			
D28 Control Cheese Core	Thermophilic Streptococci	1.47x10^{8}	2.33x10^{7}	9.93x10^{8}	8.33x10^{8}	5.7x10^{8}	7.02x10^{8}	8.83x10^{8}	1.62x10^{8}	1.47x10^{9}	5.73x10^{8}	1.17x10^{9}	4.94x10^{8}	1.28x10^{9}	3.00x10^{8}	1.54x10^{9}	3.69x10^{8}	1.41x10^{9}	3.33x10^{8}	0.046 (*)	0.077 (.)	0.562 (ns)
	Lactic bacteria	1.27x10^{9}	5.91x10^{8}	7.21x10^{8}	2.68x10^{8}	9.94x10^{8}	5.08x10^{8}	1.01x10^{9}	2.68x10^{8}	9.4x10^{8}	7.96x10^{8}	9.75x10^{8}	5.33x10^{8}	9.39x10^{8}	4.24x10^{8}	5.96x10^{8}	4.81x10^{8}	7.67x10^{8}	4.47x10^{8}	0.614 (ns)	0.102 (ns)	0.722 (ns)
	Yeasts	2.14x10^{5}	2.56x10^{5}	9.04x10^{7}	1.38x10^{8}	4.53x10^{7}	1x10^{8}	1.77x10^{5}	2.72x10^{5}	4.96x10^{6}	4.85x10^{6}	2.57x10^{6}	4.04x10^{6}	5.43x10^{4}	3.6x10^{4}	5.08x10^{5}	4.32x10^{5}	2.81x10^{5}	3.7x10^{5}	0.331 (ns)	0.003 (**)	0.335 (ns)
	Molds	5.68x10^{4}	3.89x10^{4}	9.3x10^{4}	NC[a]	6.59x10^{4}	3.66x10^{4}	5.5x10^{4}	4.06x10^{4}	2.03x10^{5}	6.72x10^{4}	1.14x10^{5}	9.21x10^{4}	4.22x10^{4}	3.87x10^{4}	2.64x10^{5}	3.06x10^{5}	1.53x10^{5}	2.3x10^{5}	0.682 (ns)	0.026 (*)	0.688 (ns)
	Ripening bacteria	2.01x10^{7}	2.24x10^{7}	1.17x10^{8}	7.55x10^{7}	6.87x10^{7}	7.29x10^{7}	9.3x10^{5}	1.18x10^{6}	1.83x10^{7}	1.53x10^{7}	9.62x10^{6}	1.36x10^{7}	7.57x10^{5}	6.31x10^{5}	7.21x10^{6}	3.19x10^{6}	3.98x10^{6}	4.09x10^{6}	0.030 (*)	0.006 (**)	0.066 (.)
D28 Control Cheese Rind	Thermophilic Streptococci	1.95x10^{8}	2.19x10^{8}	4.57x10^{8}	2.28x10^{8}	3.26x10^{8}	2.46x10^{8}	1.73x10^{7}	4.04x10^{6}	3.47x10^{8}	2.59x10^{8}	1.82x10^{8}	2.43x10^{8}	1.03x10^{8}	8.64x10^{7}	3.09x10^{8}	6.48x10^{7}	2.06x10^{8}	1.32x10^{8}	0.244 (ns)	0.003 (**)	0.828 (ns)
	Lactic bacteria	7.79x10^{8}	3.42x10^{8}	6.58x10^{8}	3.05x10^{8}	7.18x10^{8}	2.97x10^{8}	8.02x10^{8}	2.23x10^{8}	5.8x10^{8}	2.37x10^{8}	6.91x10^{8}	2.39x10^{8}	8.48x10^{8}	1.26x10^{8}	3.25x10^{8}	1.31x10^{8}	5.87x10^{8}	3.09x10^{8}	0.653 (ns)	0.011 (*)	0.354 (ns)
	Yeasts	4.03x10^{7}	1.21x10^{7}	5.2x10^{8}	4.84x10^{8}	2.8x10^{8}	4.03x10^{8}	5.87x10^{7}	1.76x10^{7}	6.48x10^{7}	1.3x10^{7}	6.18x10^{7}	1.42x10^{7}	6.33x10^{7}	7.01x10^{6}	9.28x10^{7}	3.25x10^{7}	7.81x10^{7}	2.65x10^{7}	0.673 (ns)	0.015 (*)	0.106 (ns)
	Molds	4.12x10^{7}	1.53x10^{6}	3.45x10^{7}	4.82x10^{6}	3.78x10^{7}	4.85x10^{6}	4.52x10^{7}	1.11x10^{7}	4.75x10^{7}	1.8x10^{6}	4.63x10^{7}	7.24x10^{6}	4.77x10^{7}	1.36x10^{7}	5.58x10^{7}	3.05x10^{7}	5.18x10^{7}	2.16x10^{7}	0.173 (ns)	0.667 (ns)	0.681 (ns)
	Ripening bacteria	2.74x10^{9}	7.46x10^{8}	3.8x10^{9}	1.87x10^{9}	3.27x10^{9}	1.4x10^{9}	4.56x10^{8}	1.8x10^{8}	2.75x10^{9}	6.45x10^{8}	1.6x10^{9}	1.33x10^{9}	6.71x10^{8}	4.35x10^{8}	2.53x10^{9}	9.57x10^{8}	1.6x10^{9}	1.22x10^{9}	0.119 (ns)	0.012 (*)	0.545 (ns)

Table S4. Alpha-diversity indexes calculated from the bacterial profiles in the raw milk, the cheese cores and the cheese rinds according to the farm and the lactic starter dose. The *p*-value was calculated by an ANOVA. **p* < 0.05; ***p* < 0.01; ****p* < 0.001; tendency; ns non-significant.

		Shannon's indexes						
		Farm			Dose			
Type	Time	F_15	F_38	p-value	x0.1	x1	x2	p-value
Raw milk	D0	1.71	1.85	0.105 (ns)	-	-	-	-
Cheese Core	D8	0.36	0.64	0.093 (.)	1.28	0.14	0.08	0.001 (***)
	D28	1.04	1.05	0.388 (ns)	1.64	0.86	0.64	0.001 (***)
Cheese Rind	D28	1.63	1.28	0.940 (ns)	1.81	1.56	1.00	0.086 (.)

		Observed						
		Farm			Dose			
Type	Time	F_15	F_38	p-value	x0.1	x1	x2	p-value
Raw milk	D0	20.56	13.33	0.037 (*)	-	-	-	-
Cheese Core	D8	14.22	19.11	0.032 (*)	27.17	12.33	10.50	0.005 (**)
	D28	20.78	25.78	0.019 (*)	29.67	21.00	19.17	0.185 (ns)
Cheese Rind	D28	25.11	25.89	0.325 (ns)	28.50	26.67	21.33	0.416 (ns)

		Simpson's index						
		Farm			Dose			
Type	Time	F_15	F_38	p-value	x0.1	x1	x2	p-value
Raw milk	D0	0.65	0.75	0.116 (ns)	-	-	-	-
Cheese Core	D8	0.14	0.24	0.139 (ns)	0.51	0.04	0.02	0.001 (***)
	D28	0.46	0.40	0.912 (ns)	0.67	0.36	0.26	0.001 (***)
Cheese Rind	D28	0.67	0.50	0.717 (ns)	0.74	0.63	0.39	0.039 (*)

		Chao1						
		Farm			Dose			
Type	Time	F_15	F_38	p-value	x0.1	x1	x2	p-value
Raw milk	D0	20.56	13.33	0.038 (*)	-	-	-	-
Cheese Core	D8	14.22	19.17	0.033 (*)	27.25	12.33	10.50	0.005 (**)
	D28	20.78	25.89	0.017 (*)	29.83	21.00	19.17	0.174 (ns)
Cheese Rind	D28	25.15	26.00	0.318 (ns)	28.56	26.83	21.33	0.411 (ns)

		InvSimpson's index						
		Farm			Dose			
Type	Time	F_15	F_38	p-value	x0.1	x1	x2	p-value
Raw milk	D0	3.28	5.18	0.570 (ns)	-	-	-	-
Cheese Core	D8	1.26	1.81	0.097 (.)	2.54	1.05	1.02	0.018 (*)
	D28	2.05	2.15	0.385 (ns)	3.31	1.60	1.38	0.001 (***)
Cheese Rind	D28	4.24	2.61	0.416 (ns)	4.62	3.65	2.01	0.101 (ns)

		ACE						
		Farm			Dose			
Type	Time	F_15	F_38	p-value	x0.1	x1	x2	p-value
Raw milk	D0	20.71	13.58	0.050 (.)	-	-	-	-
Cheese Core	D8	15.06	20.65	0.031(*)	27.29	12.42	11.86	0.138 (ns)
	D28	22.29	26.08	0.054 (.)	32.82	21.00	20.13	0.033 (*)
Cheese Rind	D28	25.54	26.26	0.317 (ns)	28.92	27.27	21.51	0.382 (ns)

		Fisher's index						
		Farm			Dose			
Type	Time	F_15	F_38	p-value	x0.1	x1	x2	p-value
Raw milk	D0	3.11	2.47	0.058 (.)	-	-	-	-
Cheese Core	D8	1.48	2.07	0.032 (*)	3.06	1.24	1.02	0.005 (**)
	D28	2.27	2.86	0.021 (*)	3.41	2.27	2.02	0.156 (ns)
Cheese Rind	D28	2.87	2.89	0.340 (ns)	3.26	3.03	2.35	0.432 (ns)

Table S5. Statistical assessment (*p*-values) of bacterial profiles clustering and of intra-cluster dispersion calculated according to the farm and the dose, in the raw milk, the cheese cores and the cheese rinds. The *p*-value was calculated by a pairwise Adonis test for differences between doses, and by a TukeyHSD test for the dispersion of samples. **p* < 0.05; ***p* < 0.01; ****p* < 0.001; tendency; ns non-significant.

		Bray-Curtis									
		Farm		Dose							
		Difference	Dispersion	Difference				Dispersion			
Type	Time	F_15 vs F_38	F_15 vs F_38	x0.1 vs x1	x0.1 vs x2	x1 vs x2	x0.1 vs x1 vs x2	x0.1 vs x1	x0.1 vs x2	x1 vs x2	x0.1 vs x1 vs x2
Raw milk	D0	0.001 (***)	0.017 (*)	-	-	-	-	-	-	-	-
Cheese core	D8	0.125 (ns)	0.350 (ns)	0.009 (**)	0.009 (**)	0.219 (ns)	0.002 (**)	2.11x10^{-6} (***)	1.22x10^{-6} (***)	0.929 (ns)	4.12x10^{-7} (***)
	D28	0.021 (*)	0.294 (ns)	0.017 (*)	0.009 (**)	0.245 (ns)	0.008 (**)	0.001 (***)	4.02x10^{-4} (***)	0.794 (ns)	2.59x10^{-4} (***)
Cheese rind	D28	0.289 (ns)	0.366 (ns)	0.071 (.)	0.009 (**)	0.081 (.)	0.002 (**)	0.374 (ns)	0.011 (*)	0.150 (ns)	0.014 (*)

		Jaccard									
		Farm		Dose							
		Difference	Dispersion	Difference				Dispersion			
Type	Time	F_15 vs F_38	F_15 vs F_38	x0.1 vs x1	x0.1 vs x2	x1 vs x2	x0.1 vs x1 vs x2	x0.1 vs x1	x0.1 vs x2	x1 vs x2	x0.1 vs x1 vs x2
Raw milk	D0	0.001 (***)	0.017 (*)	-	-	-	-	-	-	-	-
Cheese core	D8	0.133 (ns)	0.350 (ns)	0.003 (**)	0.003 (**)	0.215 (ns)	0.007 (**)	2.11x10^{-6} (***)	1.22x10^{-6} (***)	0.929 (ns)	4.12x10^{-7} (***)
	D28	0.035 (*)	0.294 (ns)	0.012 (*)	0.009 (**)	0.256 (ns)	0.012 (*)	0.001 (***)	4.02x10^{-4} (***)	0.794 (ns)	2.59x10^{-4} (***)
Cheese rind	D28	0.279 (ns)	0.366 (ns)	0.075 (.)	0.006 (**)	0.078 (.)	0.001 (***)	0.373 (ns)	0.011 (*)	0.150 (ns)	0.014 (*)

		Weighted Unifrac									
		Farm		Dose							
		Difference	Dispersion	Difference				Dispersion			
Type	Time	F_15 vs F_38	F_15 vs F_38	x0.1 vs x1	x0.1 vs x2	x1 vs x2	x0.1 vs x1 vs x2	x0.1 vs x1	x0.1 vs x2	x1 vs x2	x0.1 vs x1 vs x2
Raw milk	D0	0.001 (***)	0.006 (**)	-	-	-	-	-	-	-	-
Cheese core	D8	0.062 (.)	0.233 (ns)	0.009 (**)	0.009 (**)	0.237 (ns)	0.012 (*)	5.86x10^{-5} (***)	3.88x10^{-5} (***)	0.970 (ns)	1.44x10^{-5} (***)
	D28	0.073 (.)	0.246 (ns)	0.011 (*)	0.011 (*)	0.246 (ns)	0.011 (*)	0.002 (**)	0.004 (**)	0.935 (ns)	0.001 (***)
Cheese rind	D28	0.309 (ns)	0.613 (ns)	0.039 (*)	0.006 (**)	0.085 (.)	0.002 (**)	0.832 (ns)	0.054 (.)	0.152 (ns)	0.053 (.)

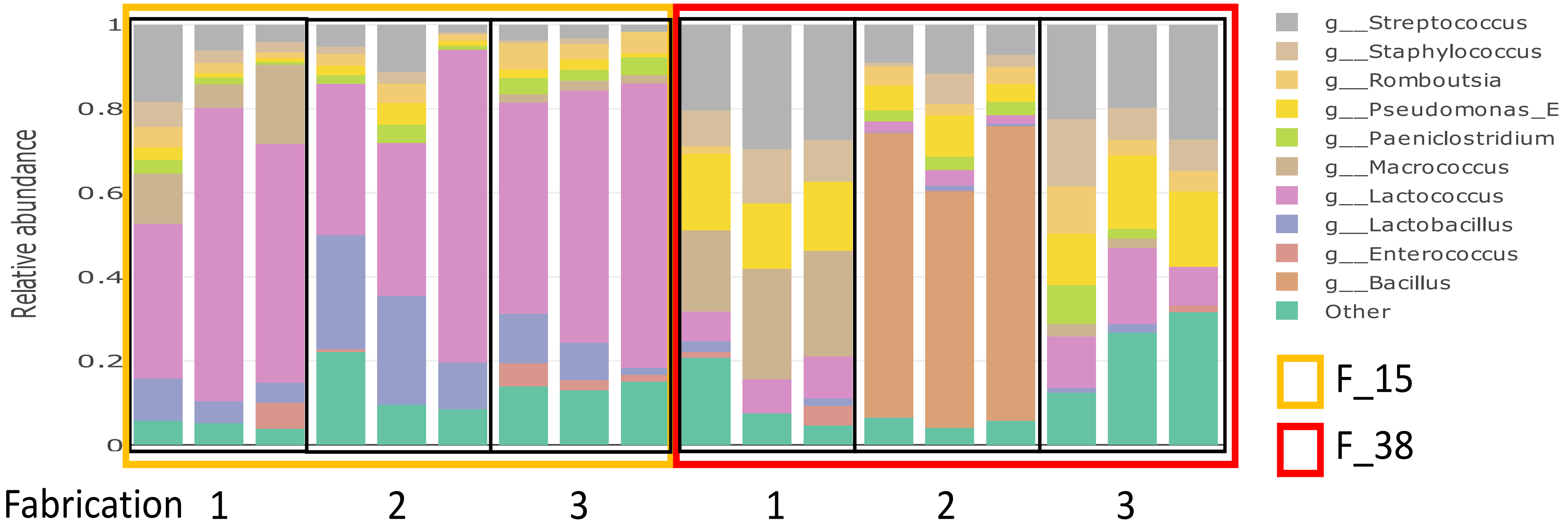

Figure S1. Relative abundance of the 10 major genera detected in the raw milk, organized by farms and days of cheese-making.

Table S6. Mean relative abundance for taxa differentially abundant between tested conditions (farm or lactic starter dose). UD: undetected. NA: not available because of no starter in raw milk.

	Family	Farm			Dose			
		F_15	F_38	difference	x0.1	x1	x2	difference x0.1 vs
Milks	f__Bacillaceae	0.000	0.216	yes	NA	NA	NA	NA
Milks	f__Bifidobacteriaceae	0.017	0.000	yes	NA	NA	NA	NA
Milks	f__Corynebacteriaceae	0.017	0.000	yes	NA	NA	NA	NA
Milks	f__Lactobacillaceae	0.145	0.019	yes	NA	NA	NA	NA
Milks	f__Pseudomonadaceae	0.021	0.135	yes	NA	NA	NA	NA
Milks	f__Streptococcaceae	0.608	0.282	yes	NA	NA	NA	NA
D8 Core	f__Enterobacteriaceae	0.007	0.033	no	0.071	0.001	0.001	x1 & x2
D8 Core	f__Enterococcaceae	0.001	0.020	no	0.036	0.001	0.000	x2
D8 Core	f__Lactobacillaceae	0.005	0.001	yes	0.004	0.003	0.002	no
D8 Core	f__Streptococcaceae	0.982	0.925	no	0.843	0.990	0.995	x1 & x2
D28 Core	f__Enterococcaceae	0.003	0.036	yes	0.045	0.008	0.004	no
D28 Core	f__Streptococcaceae	0.783	0.838	no	0.709	0.828	0.863	x1 & x2
D28 Rind	f__Aerococcaceae	0.001	UD	no	0.002	UD	0.000	x2
D28 Rind	f__Enterobacteriaceae	0.089	0.274	no	0.341	0.136	0.043	x2
D28 Rind	f__Staphylococcaceae	0.022	0.003	no	0.035	0.004	0.001	x2
D28 Rind	f__Streptococcaceae	0.649	0.546	no	0.235	0.647	0.878	x1 & x2

	Genus	Farm			Dose			
		F_15	F_38	difference	x0.1	x1	x2	difference x0.1 vs
Milks	g__Bifidobacterium	0.017	0.000	yes	NA	NA	NA	NA
Milks	g__Lacticaseibacillus	0.022	0.007	yes	NA	NA	NA	NA
Milks	g__Lactobacillus	0.119	0.011	yes	NA	NA	NA	NA
Milks	g__Lactococcus	0.545	0.083	yes	NA	NA	NA	NA
Milks	g__Pseudomonas_E	0.021	0.136	yes	NA	NA	NA	NA
Milks	g__Streptococcus	0.061	0.200	yes	NA	NA	NA	NA
D8 Core	g__Enterobacteriaceae_genus	0.006	0.007	no	0.024	0.001	0.000	x1 & x2
D8 Core	g__Enterococcus	0.001	0.020	no	0.037	0.001	0.000	x2
D8 Core	g__Lacticaseibacillus	0.004	0.001	yes	0.004	0.001	0.001	no
D8 Core	g__group_Lactobacillus_Lentilactobacillus	0.001	0.001	no	0.000	0.001	0.001	x2
D8 Core	g__Lactococcus	0.044	0.058	no	0.170	0.007	0.002	x1 & x2
D8 Core	g__Staphylococcus	0.001	0.008	yes	0.013	0.001	UD	no
D8 Core	g__Streptococcus	0.926	0.861	no	0.648	0.978	0.991	x1 & x2
D28 Core	g__Brevibacterium	0.004	0.003	no	0.009	0.001	0.001	x1
D28 Core	g__Enterobacteriaceae_genus	0.005	0.003	no	0.011	0.002	0.001	x2
D28 Core	g__Enterococcus	0.003	0.036	yes	0.046	0.008	0.004	no
D28 Core	g__Lactococcus	0.049	0.087	no	0.208	0.024	0.008	x1 & x2
D28 Core	g__Serratia	0.000	0.018	yes	UD	UD	UD	no
D28 Core	g__Staphylococcus	UD	0.001	no	0.001	0.000	UD	x1
D28 Core	g__Streptococcus	0.715	0.738	no	0.466	0.789	0.850	x1 & x2
D28 Rind	g__Aerococcus	0.001	UD	no	0.002	UD	0.000	x2
D28 Rind	g__Brevibacterium	0.182	0.061	no	0.259	0.109	0.041	x2
D28 Rind	g__Macrococcus	0.004	0.002	no	0.008	UD	0.000	x2
D28 Rind	g__Staphylococcus	0.005	0.001	no	0.008	0.001	0.000	x2
D28 Rind	g__Streptococcaceae_genus	0.043	0.012	no	0.007	0.033	0.041	x2
D28 Rind	g__Streptococcus	0.420	0.451	no	0.048	0.453	0.753	x1 & x2

	Species	Farm			Dose			
		F_15	F_38	difference	x0.1	x1	x2	difference x0.1 vs
Milks	s__Bifidobacterium_mongoliense	0.017	0.000	yes	NA	NA	NA	NA
Milks	s__Lacticaseibacillus_group_casei_paracasei_zeae	0.022	0.007	yes	NA	NA	NA	NA
Milks	s__Lactobacillus_group_crispatus_gallinarum_helveticus	0.114	0.000	yes	NA	NA	NA	NA
Milks	s__Lactococcus_lactis	0.547	0.084	yes	NA	NA	NA	NA
Milks	s__Pseudomonas_group_canadensis_fluorescens	0.021	0.136	yes	NA	NA	NA	NA
D8 Core	s__Enterobacteriaceae_species	0.006	0.007	no	0.024	0.001	0.000	x1 & x2
D8 Core	s__Enterococcus_faecalis	0.001	0.022	yes	0.034	0.001	UD	no
D8 Core	s__Lacticaseibacillus_group_casei_paracasei_zeae	0.004	0.001	yes	0.004	0.001	0.001	no
D8 Core	s__Lactobacillus_delbrueckii	0.001	0.001	no	0.000	0.001	0.001	x2
D8 Core	s__Lentilactobacillus_parabuchneri	0.000	0.000	yes	UD	0.000	0.000	no
D8 Core	s__Lactococcus_lactis	0.044	0.058	no	0.168	0.007	0.002	x1 & x2
D8 Core	s__Staphylococcus_xylosus	0.001	0.008	yes	0.013	0.001	UD	no
D8 Core	s__Streptococcus_thermophilus	0.926	0.864	no	0.649	0.978	0.991	x1 & x2
D28 Core	s__Brevibacterium_group_ammoniilyticum_aurantiacum_marinum	0.001	0.002	no	0.007	0.001	0.001	x1
D28 Core	s__Enterobacteriaceae_species	0.005	0.003	no	0.011	0.002	0.001	x2
D28 Core	s__Enterococcus_faecalis	0.001	0.026	yes	0.035	0.004	0.001	no
D28 Core	s__Lactobacillus_delbrueckii	0.001	0.001	no	0.000	0.001	0.001	x1
D28 Core	s__Lactococcus_lactis	0.049	0.087	no	0.207	0.021	0.008	x2
D28 Core	s__Serratia_group_grimesii_liquefaciens	0.000	0.018	yes	0.015	0.001	UD	no
D28 Core	s__Staphylococcus_xylosus	UD	0.001	no	0.001	UD	UD	x1
D28 Core	s__Streptococcus_thermophilus	0.715	0.738	no	0.466	0.800	0.850	x1 & x2
D28 Rind	s__Brevibacterium_group_antiquum_aurantiacum	0.050	0.015	no	0.071	0.028	0.011	x2
D28 Rind	s__Brevibacterium_group_ammoniilyticum_aurantiacum_marinum	0.133	0.045	no	0.190	0.081	0.030	x2
D28 Rind	s__Streptococcus_group_loxodontisalivarius_saliviloxodontae	0.043	0.012	no	0.007	0.032	0.041	x1 & x2
D28 Rind	s__Streptococcus_thermophilus	0.420	0.452	no	0.048	0.453	0.754	x1 & x2
